# Identification of plant vacuolar transporters mediating phosphate storage

**DOI:** 10.1038/ncomms11095

**Published:** 2016-03-31

**Authors:** Tzu-Yin Liu, Teng-Kuei Huang, Shu-Yi Yang, Yu-Ting Hong, Sheng-Min Huang, Fu-Nien Wang, Su-Fen Chiang, Shang-Yueh Tsai, Wen-Chien Lu, Tzyy-Jen Chiou

**Affiliations:** 1 Agricultural Biotechnology Research Center, Academia Sinica, No. 128, Academia Road, Section 2, Taipei 11529, Taiwan; 2 Molecular and Biological Agricultural Sciences Program, Taiwan International Graduate Program, Academia Sinica, Taipei 11529, Taiwan; 3 Graduate Institute of Biotechnology, National Chung-Hsing University, Taichung 40243, Taiwan; 4 Department of Biomedical Engineering and Environmental Sciences, National Tsing Hua University, Hsinchu 30013, Taiwan; 5 Graduate Institute of Applied Physics, National Chengchi University, Taipei 11605, Taiwan; 6 Biotechnology Center, National Chung-Hsing University, Taichung 40243, Taiwan

## Abstract

Plant vacuoles serve as the primary intracellular compartments for inorganic phosphate (Pi) storage. Passage of Pi across vacuolar membranes plays a critical role in buffering the cytoplasmic Pi level against fluctuations of external Pi and metabolic activities. Here we demonstrate that the SPX-MFS proteins, designated as PHOSPHATE TRANSPORTER 5 family (PHT5), also named Vacuolar Phosphate Transporter (VPT), function as vacuolar Pi transporters. Based on ^31^P-magnetic resonance spectroscopy analysis, *Arabidopsis pht5;1* loss-of-function mutants accumulate less Pi and exhibit a lower vacuolar-to-cytoplasmic Pi ratio than controls. Conversely, overexpression of PHT5 leads to massive Pi sequestration into vacuoles and altered regulation of Pi starvation-responsive genes. Furthermore, we show that heterologous expression of the rice homologue OsSPX-MFS1 mediates Pi influx to yeast vacuoles. Our findings show that a group of Pi transporters in vacuolar membranes regulate cytoplasmic Pi homeostasis and are required for fitness and plant growth.

## Abstract (web summary)

The plant vacuole acts as a storage compartment for inorganic phosphate and buffers cytoplasmic phosphate concentration. Here, Liu *et al*. identify a group of vacuolar phosphate transporters in *Arabidopsis* that are required for plant growth in response to fluctuating availability of phosphate.

## Graphical abstract

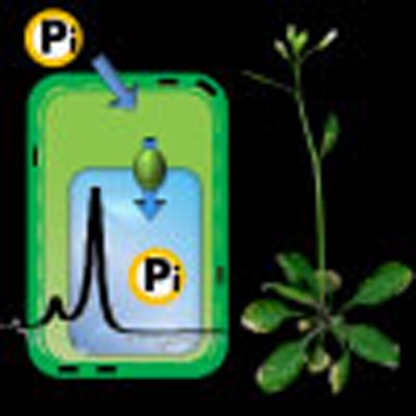

Phosphorus (P) acquired in the form of inorganic phosphate (Pi) is one of the most abundant macronutrients in plant tissues^1^,^2^. Owing to its chemical properties, Pi forms insoluble complexes or precipitates with organic matter or mineral cations that are easily immobilized in the soil, rendering Pi availability a limiting factor for plant growth and development^3^. To ensure crop productivity, farmers apply large quantities of Pi fertilizers produced from non-renewable rock phosphate. Concern over the gradual depletion of global P reserves and the increasing demand for high crop yields due to increasing world population over the past few decades has resulted in a need to better understand how to develop crop varieties that use Pi more efficiently, thereby cutting down the huge costs incurred by fertilizer consumption and providing a means to achieve sustainable agriculture^4^.

Grown under varying conditions of Pi availability, plants coordinate discrete Pi transport activities across membranes, to maintain the cellular Pi homeostasis required for metabolic regulation and signal transduction^5^. For example, members of the PHOSPHATE TRANSPORTER 1 (PHT1) family, localized in the plasma membranes, are responsible for the external Pi acquisition and/or Pi translocation between cells or tissues^6^, whereas the members of the PHT2, PHT3 and PHT4 families belong to the organelle Pi transporters, targeted to mitochondria, plastids or Golgi for energy metabolism and stress responses^7^,^8^,^9^,^10^,^11^,^12^.

The vacuole, which occupies most of the volume of the plant cell, serves as a primary intracellular compartment for storage and remobilization of Pi^13^. Under adequate Pi supply, ∼70–95% of the intracellular Pi is stored in the vacuole^14^. When external Pi is in scarce supply, the level of cytoplasmic Pi (cyt-Pi; the sum of Pi in the cytosol and the non-vacuole organelles) is kept relatively constant at the expense of vacuolar Pi (vac-Pi) (refs 15, 16), whereas when Pi is resupplied to Pi-starved plants, rapid and massive Pi uptake to the plasma membrane is accompanied by the efficient sequestration of Pi inside the vacuole^17^,^18^. Despite the critical role of vac-Pi in buffering the cyt-Pi against fluctuations caused by variable Pi availability and metabolic activities, the molecular identity of the vac-Pi transporter as well as the regulatory mechanism by which Pi is translocated across vacuolar membranes (tonoplast) remain elusive.

Several eukaryotic SYG1/PHO81/XPR1 (SPX) domain-containing proteins have been implicated in the regulation of Pi signalling and transport^19^,^20^, including the yeast low-affinity Pi transporters Pho87, Pho90 and Pho91 (refs 21, 22). In plants, SPX domain-containing proteins can be classified into different families based on additional domains at the carboxyl terminus^23^,^24^. One of them possesses the major facilitator superfamily (MFS) domain that is found in many transporters mediating the translocation of small solutes, including Pi^25^. We therefore envisaged that the plant SPX-MFS family potentially functions as a new group of Pi transporters in plants.

Although the three *Arabidopsis* SPX-MFS proteins (At1g63010, At4g11810 and At4g22990) share only low sequence similarity (below 25%) with the other known *Arabidopsis* Pi transporters, the capability of SPX-MFS proteins to transport Pi was assumed based on the observations of partial complementation of the Pi uptake-defective yeast mutants by the rice OsSPX-MFS1 or OsSPX-MFS3 and heterologous expression of OsSPX-MFS3 at the plasma membranes of *Xenopus* oocytes^26^,^27^. However, their transport properties and physiological roles in plants are unclear. In this study, we identified the *Arabidopsis* SPX-MFS proteins as the transporters mediating vac-Pi storage. Using *in vivo*^31^P-magnetic resonance spectroscopy (MRS) analysis, we demonstrate that *AtSPX-MFS1* loss-of-function mutants exhibit a lower vac/cyt-Pi ratio than wild-type (WT) plants, whereas overexpression of *AtSPX-MFS* genes leads to misregulation of Pi starvation-responsive (PSR) genes and growth retardation as a consequence of massive Pi sequestration into vacuoles. We also show the capability of the rice homologue OsSPX-MFS1 to mediate Pi influx into yeast vacuoles. The *Arabidopsis* SPX-MFS proteins are thus designated here as members of the PHOSPHATE TRANSPORTER 5 family (PHT5) following the systematic nomenclature of PHT1–PHT4 Pi transporters in *Arabidopsis*^28^,^29^. PHT5;1 was also named Vacuolar Phosphate Transporter 1 (VPT1) in a very recent article^30^. Our findings shed light on the crucial role of SPX-MFS in plant adaptation to Pi fluctuations and provide a new direction to explore the mechanisms by which plants modulate cyt-Pi homeostasis in response to varying Pi availability through understanding of the regulation of vac-Pi transporters.

## Results

### *Arabidopsis* PHT5 proteins reside in the vacuolar membrane

**Figure 1:**
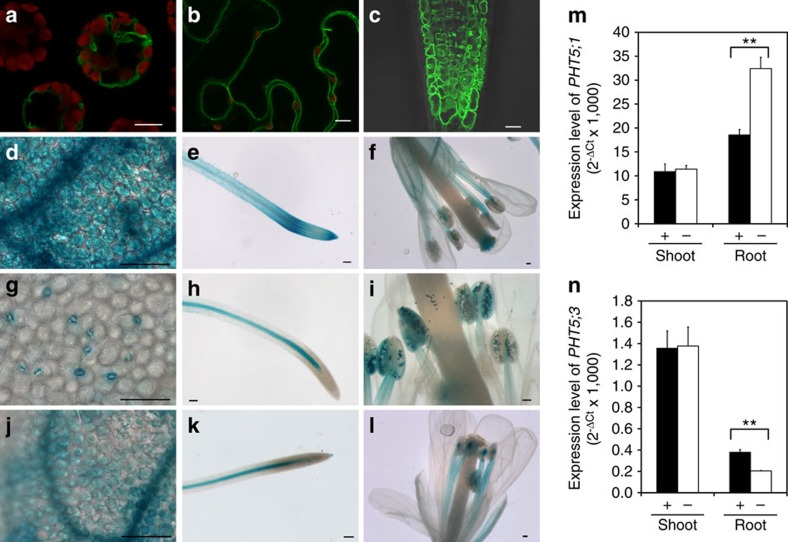
Expression and tonoplast localization of AtPHT5 family members. Expression of *p35S:PHT5;1-GFP* in *Arabidopsis* mesophyll protoplasts (**a**), tobacco (*N. benthamiana*) leaves (**b**) and roots of *Arabidopsis* transgenic plants (**c**). Expression of *pPHT5;1:GUS* (**d**–**f**), *pPHT5;2:GUS* (**g**–**i**) and *pPHT5;3:GUS* (**j**–**l**) in 12-day-old seedlings grown under Pi-sufficient conditions (**d**,**e**,**g**,**h**,**j**,**k**) and in flowers of transgenic plants grown in soils (**f**,**i**,**l**). Scale bars, 10 μm (**a**,**b**,**c**); 100 μm (**d**–**l**). qRT–PCR analysis of *PHT5;1* (**m**) and *PHT5;3* (**n**) expression in the shoot and root of 13-day-old WT seedlings under Pi-sufficient (+) and Pi-deficient (–, 7 days of Pi starvation) conditions. Error bar, s.e. (*n*=3). **P<0.01, Student's *t*-test. Results of qRT–PCR were reproducible in three independent experiments.

The *Arabidopsis* PHT5 family consists of three members, namely *At*PHT5;1 (At1g63010), *At*PHT5;2 (At4g11810) and *At*PHT5;3 (At4g22990). To characterize their function, we first determined the subcellular localization of these proteins by fusing them with green fluorescent protein (GFP) and expressing them in *Arabidopsis* mesophyll protoplasts (Fig. 1a and Supplementary Fig. 1a,b), tobacco (*Nicotiana benthamiana*) leaves (Fig. 1b and Supplementary Fig. 1c,d) and *Arabidopsis* plants (Fig. 1c) under the control of the cauliflower mosaic virus 35S promoter. The fluorescent labelling of all three GFP fusion proteins coincided with the tonoplast. The observation of the tonoplast localization of these proteins with potential as transporters prompted us to postulate that they might be the long-sought-after vac-Pi transporters. Further analyses of promoter activities using the β-glucuronidase (GUS) reporter system indicated that the three *PHT5* genes displayed distinct but partially overlapping expression patterns. *PHT5;1* was ubiquitously expressed in most tissues (Fig. 1d–f) and *PHT5;2* expression was confined to guard cells, vascular tissue and pollen (Fig. 1g–i). *PHT5;3* showed a similar expression pattern to *PHT5;1*, but in the root its expression was exclusive to the stele (Fig. 1j–l). The results of quantitative reverse transcription–PCR (qRT–PCR) showed that the *PHT5;1* transcript was the most abundant among the gene family and the expression level of *PHT5;2* was the lowest and hardest to detect. In shoots, the level of *PHT5;1* and *PHT5;3* transcript was independent of Pi status (Fig. 1m,n). In roots, although *PHT5;1* was upregulated in response to Pi deficiency, *PHT5;3* was downregulated (Fig. 1m,n). Consistent with these results, the differences in the expression level of *PHT5* genes were also observed in the subsequent RNA sequencing (RNA-seq) analyses (Supplementary Data 1).

### Loss of *PHT5;1* leads to impaired Pi accumulation

**Figure 2:**
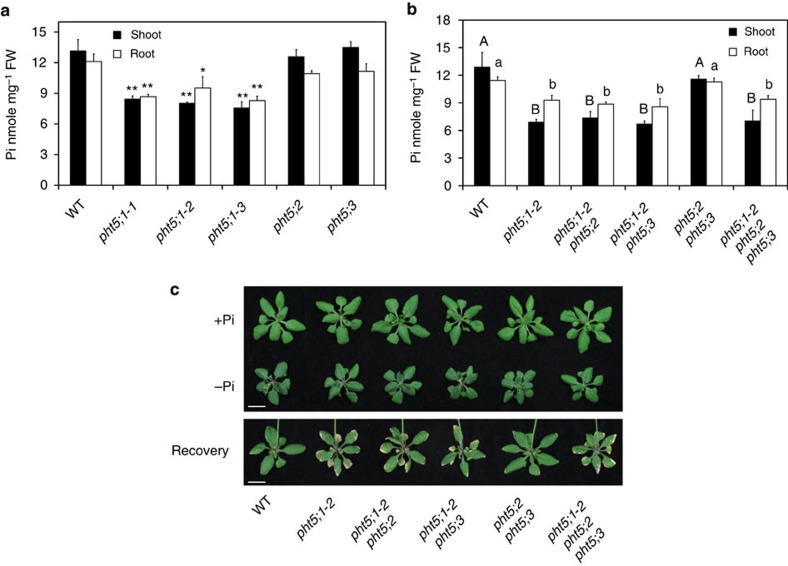
The capacity of Pi storage and recovery is impaired in the *pht5;1* mutants. The Pi level of WT, *pht5;1-1*, *pht5;1-2*, *pht5;1-3, pht5;2* and *pht5;3* plants (**a**, asterisks indicate a significant difference compared with WT, *P<0.05, **P<0.01, Student's *t*-test) and their respective double and triple mutants (**b**, different upper and lower case letters represent a significant difference among genotypes in the shoot and root samples, respectively, analysis of variance *P*<0.05) grown under Pi-sufficient conditions. The phenotype of 21-day WT, *pht5;1-2*, *pht5;1-2 pht5;2*, *pht5;1-2 pht5;3*, *pht5;2 pht5;3* and *pht5;1-2 pht5;2 pht5;3* plants grown under Pi-sufficient (+P) and Pi-deficient (−Pi) conditions, and after 3 days of Pi replenishment following 3 days of Pi starvation (Recovery) (**c**). Error bar, s.d. (*n*=3). Scale bars, 10 mm. Results were reproducible in at least three independent experiments.

We then analysed the *Arabidopsis* T-DNA insertion lines for the three respective *PHT5* genes (Supplementary Fig. 2a–d). Under Pi-sufficient conditions, the *pht5;1-1* knockdown mutant, as well as the *pht5;1-2* and *pht5;1-3* knockout mutants, led to a decrease in Pi levels up to 40% compared with the WT plants (Fig. 2a). By contrast, neither the *pht5;2* nor the *pht5;3*, nor the *pht5;2 pht5;3* double mutants showed an altered Pi level (Fig. 2a,b), suggesting that PHT5;1 plays a prominent role in Pi accumulation. Likewise, the double and the triple mutants lacking *PHT5;1* but not the *pht5;2 pht5;3* double mutant showed a reduced Pi level (Fig. 2b). The phenotype of *pht5;1* mutants could be restored by the expression of *PHT5;1* driven by the native promoter (Supplementary Fig. 2e), indicating that the reduction of the Pi level resulted from the loss of *PHT5;1*.

As the majority of the cellular Pi is stored in the vacuole, the reduction of the Pi level in the *pht5;1* mutant can probably be attributed to the decreased accumulation of vac-Pi. We then investigated whether PHT5 family members mediate the sequestration of the bulk of cyt-Pi during Pi replenishment. The loss-of-function mutants of *PHT5;1* (*pht5;1-2*, *pht5;1-2 pht5;2*, *pht5;1-2 pht5;3* and *pht5;1-2 pht5;2 pht5;3*) developed severe leaf necrosis during Pi recovery following deficiency (Fig. 2c). As massive flux of Pi is transported into the cell on Pi replenishment, an efficient sequestration of Pi into the vacuole would be essential for the maintenance of cyt-Pi level in a physiological range. It is likely that loss of *PHT5;1* impairs such activity, leading to the accumulation of cyt-Pi up to a toxic level and thus the necrosis at leaf margins as a symptom of Pi toxicity^31^. These observations suggest that PHT5;1 has an important role in adaptation to Pi fluctuations.

### Overexpression of *PHT5* leads to Pi overaccumulation

**Figure 3:**
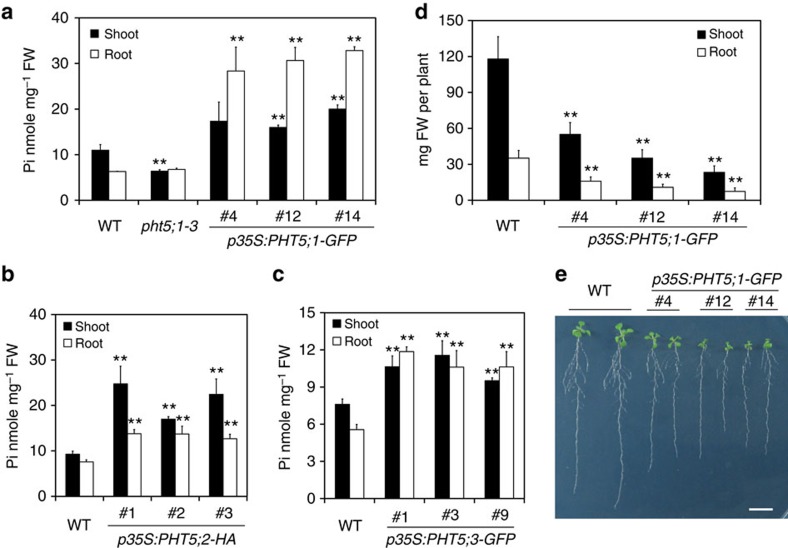
Overexpression of *PHT5* leads to Pi overaccumulation and retarded growth. The Pi level of the shoots and roots of *PHT5-*overexpressing lines grown under Pi-sufficient conditions. *p35S:PHT5;1-GFP* in **a**, *p35S:PHT5;2-HA* in **b** and *p35S:PHT5;3-GFP* in **c**. The fresh weight of 21-day-old seedlings (**d**) and the growth phenotypes of 11-day-old seedlings (**e**) of *PHT5;1-GFP-*overexpressing lines. Scale bar, 1 cm. Three independent transgenic lines are shown for each construct. Error bar, s.d. (*n*=3). Asterisks indicate a significant difference compared with WT, *P<0.05 and **P<0.01, Student's *t*-test. Results were reproducible in at least three independent experiments.

The results from the analysis of *pht5;1* mutants suggested a potential role for PHT5 in mediating Pi import to the vacuole. To further examine this possibility, we analysed the transgenic plants overexpressing PHT5 proteins. In contrast to the *pht5;1* mutant lines, the overexpression of *PHT5;1-GFP* resulted in Pi overaccumulation (Fig. 3a). The *PHT5;2-HA*- or *PHT5;3-GFP*-overexpressing lines also exhibited a higher level of Pi (Fig. 3b,c), indicating that the PHT5 members function similarly. In addition, the *PHT5;1-GFP*-overexpressing lines, as well as *PHT5;2-HA-* and *PHT5;3-GFP*-overexpressors, showed retarded growth as reflected in an ∼50% reduction of the fresh weight compared with WT plants (Fig. 3d,e and Supplementary Fig. 3). We inferred that the overexpression of PHT5 proteins sequesters Pi, thus stopping it from being used in the cytoplasm and leading to stunted growth.

### PHT5 mediates Pi influx into vacuoles

**Figure 4:**
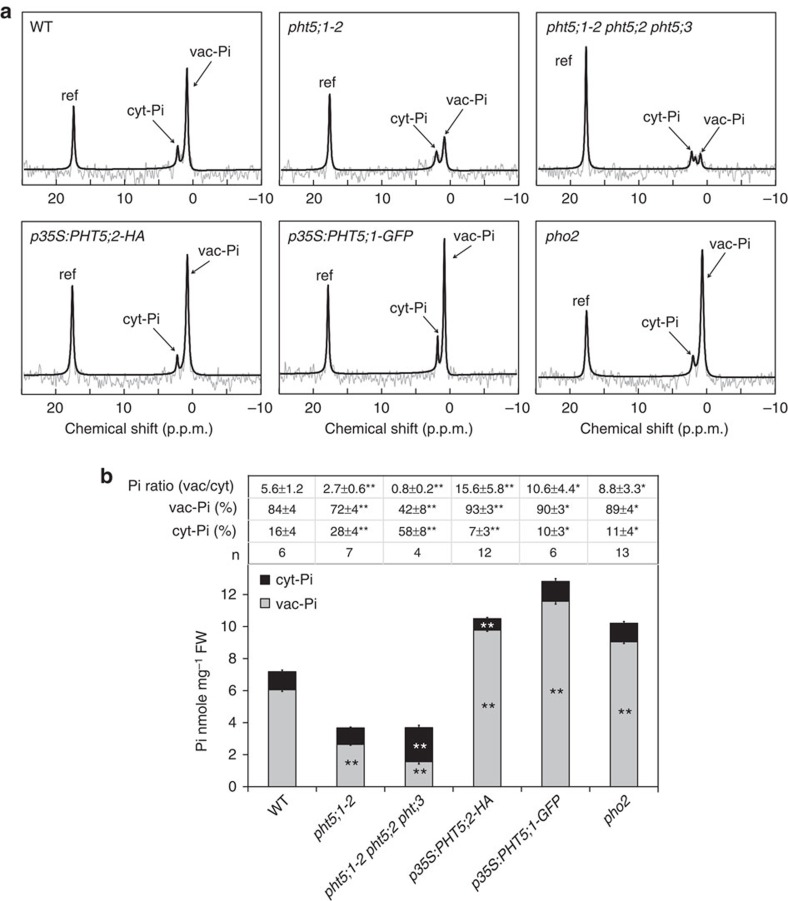
PHT5 proteins regulate the storage of vac-Pi and cyt-Pi homeostasis. ^31^P-MRS analysis of vac-Pi and cyt-Pi (**a**), Pi distribution between the vacuole and the cytoplasm, and the calculated vac-Pi and cyt-Pi levels (**b**) of WT, *pht5;1-2*, *pht5;1-2 pht5;2 pht5;3*, *p35S:PHT5;2-HA, p35S:PHT5;1-GFP* and *pho2* seedlings grown under Pi-sufficient conditions. ref, methylene diphosphonate (MDP). Asterisks indicate a significant difference compared with WT, *P<0.05 and **P<0.01, Student's *t*-test, ±s.d.; error bar, s.e. The numbers of biological replicates (*n*) are indicated in **b**. Results were combined from three to six independent experiments.

To address whether PHT5 proteins directly contribute to the vacuolar storage of Pi, we applied ^31^P-MRS-based analysis associated with magnetic resonance image. This technique, which was adapted from nuclear magnetic resonance (NMR) and is capable of differentiating between the vac-Pi and cyt-Pi pools by a chemical shift, owing to the different pH environments^32^, allows the non-invasive analysis of intact living plants without the circulating perfusion system. After ∼1 h of scan time, the two resolvable peaks corresponding to vac-Pi and cyt-Pi in the *Arabidopsis* WT whole seedlings were resolved at 1.1±0.09 p.p.m. (±s.d., *n*=6) and 2.4±0.11 p.p.m. (*n*=6), respectively (Fig. 4a). The vac-Pi and cyt-Pi levels of the *pht5;1-2*, the triple mutants, the *PHT5* overexpressors and the Pi overaccumulator *pho2* mutant^33^,^34^ were inspected under the same conditions (Fig. 4a). The vac/cyt Pi ratio of WT seedlings was calculated to be 5.6±1.2 (*n*=6), referring the distribution of 84% of Pi in the vacuole and 16% of Pi in the cytoplasm (Fig. 4b). Although the *pht5;1-2* exhibited a lower ratio of vac/cyt Pi (2.7±0.6) (±s.d., *n*=7) relative to WT plants, the *PHT5* overexpressor had a higher ratio (15.6±5.8 for PHT5;2-HA, *n*=12; 10.6±4.4 for PHT5;1-GFP, *n*=6; Fig. 4b). Remarkably, the vac-Pi peak of the triple mutant was nearly abolished with an extremely low vac/cyt Pi (0.8±0.2, *n*=4). These results suggest that PHT5 proteins regulate the vacuolar compartmentation of Pi and are functionally redundant.

Using the same batch of seedlings subjected to MRS analysis, in parallel we measured the total Pi level of the plants and calculated the subcellular Pi distribution between the vacuoles and cytoplasm for each mutant and overexpression line (Fig. 4b). Both the *pht5;1-2* and the triple mutants had a reduced vac-Pi level, but an increased cyt-Pi level was only observed for the triple mutant. By contrast, the *PHT5*-overexpressing lines displayed an increased vac-Pi level, but the *PHT5;2-HA*-overexpressing lines exhibited a decreased cyt-Pi level. Although the *pho2* mutant also exhibited a higher ratio of vac/cyt Pi (8.8±3.3, *n*=13, Fig. 4b), the cyt-Pi level of the *pho2* mutant was not significantly different from that of the WT plants (Fig. 4b), suggesting that *pho2* is able to maintain Pi homeostasis at the cellular level by increasing the capacity of vacuolar transport of Pi at this growth stage. Taken together, these results reinforce the notion that PHT5 proteins mediate the vacuolar storage of Pi and play a crucial role in the maintenance of cyt-Pi concentrations.

**Figure 5:**
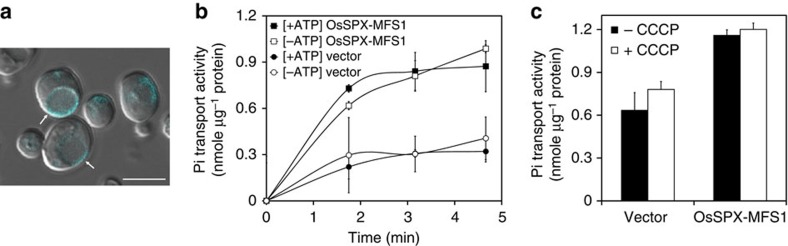
Phosphate transport activity of rice OsSPX-MFS1 in isolated yeast vacuoles. (**a**) Fluorescence signal of yeast *vtc4* mutant transformed with *OsSPX-MFS1-ECFP*. Arrows indicate the appearance of fluorescence signals in the tonoplast of yeast. Scale bar, 5 μm. ^33^Pi uptake activity of vacuoles isolated from yeast *vtc4* mutants transformed with empty vector (pAG415GPD) or expressing *OsSPX-MFS1* in the presence of 4 mM ATP [+ATP] or the absence of ATP [-ATP] at different time points (**b**) or in the presence of 10 μM carbonyl cyanide m-chlorophenyl hydrazone (CCCP) [+CCCP] or the absence of CCCP [−CCCP] at 285 s post incubation (**c**). Error bar, s.e. (*n*=3). Results were reproducible in two to four independent experiments.

To demonstrate the ability of PHT5 proteins to transport Pi, we expressed *AtPHT5;1* in the yeast (*Saccharomyces cerevisiae*) *vtc4* mutant defective in the synthesis and the vacuolar transport of polyphosphate (polyP)^35^,^36^. We did not detect differences in the Pi transport activity of the isolated vacuoles between the cells expressing *AtPHT5;1* and the empty vector controls. This was probably due to the mislocalization or the rapid degradation of the *At*PHT5;1-ECFP fusion proteins, as the fluorescence signal was mostly detected inside the vacuole. By contrast, when the rice *PHT5;1* homologue (*OsSPX-MFS1*, Os04g48390) was expressed, the localization of OsSPX-MFS1-ECFP was clearly confined to the tonoplast of yeast cells (Fig. 5a), indicating a proper vacuolar targeting of *OsSPX-MFS1*. We next isolated vacuoles from the *vtc4* mutant expressing *OsSPX-MFS1* for Pi uptake assays. After incubation in the ^33^P-labelled Pi medium, the vacuoles isolated from *OsSPX-MFS1*-expressing cells exhibited an increased uptake activity of Pi compared with the empty vector controls, regardless of the presence or the absence of ATP (Fig. 5b). Moreover, the Pi uptake activities were not abolished by the proton ionophore, carbonyl cyanide m-chlorophenyl hydrazone (Fig. 5c). These results indicate that OsSPX-MFS1 facilitates the Pi import to the yeast vacuole independent of ATP and H^+^ gradient.

Similar to the AtPHT5 proteins, GFP-OsSPX-MFS1 also showed a tonoplast localization in *Arabidopsis* and it restored the low Pi level to the WT level when expressed in *pht5;1-2* or *pht5;1-3* mutants (Supplementary Fig. 4a,b), suggesting that the *Arabidopsis* PHT5 proteins and the rice SPX-MFS1 have a similar function in facilitating Pi transport into the plant vacuole.

### Altered PHT5 expression disturbs the expression of PSR genes

To evaluate the impact of altered expression of PHT5 family members on Pi starvation responses, we checked the expression level of a number of known Pi starvation-induced (PSi) genes in the *pht5;1-2*, the triple mutants, and two independent PHT5;1-overexpressors by qRT–PCR (Supplementary Fig. 5). Nearly all of the PSi genes examined were upregulated in the *PHT5;1* overexpressors grown under Pi-sufficient conditions. By contrast, these PSi genes were downregulated in the triple mutants, but to a lesser extent in comparison with the *PHT5;1* overexpressors. We therefore focused on the *PHT5;1* overexpressors and analysed the transcriptome by RNA-seq analyses (Supplementary Data 1). Two independent *PHT5;1-GFP*-overexpressing lines 4 and 12 were examined.

**Figure 6:**
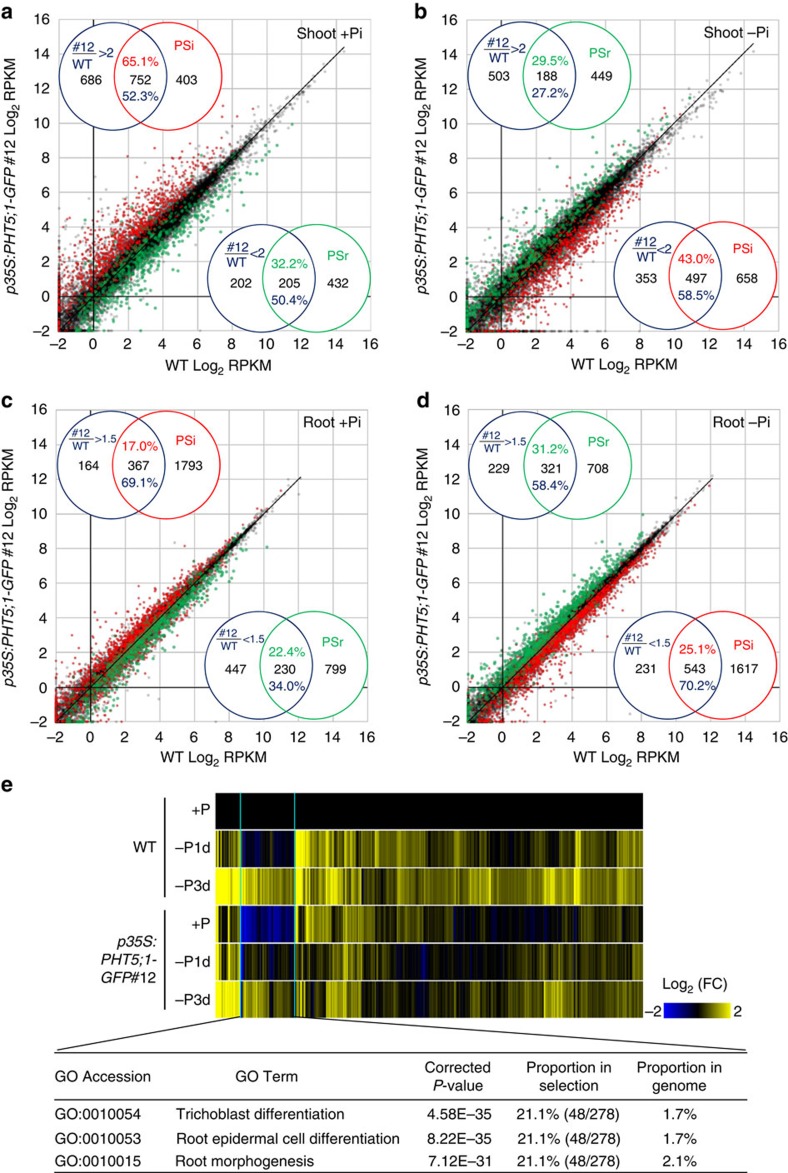
Overexpression of *PHT5;1* leads to misregulation of PSR genes. Scatter plots show the comparison of the gene expression (RPKM) in the shoot (**a**,**b**) and root (**c**,**d**) between the *PHT5;1-GFP*-overexpressing line #12 and WT plants under Pi-sufficient (+Pi) (**a**,**c**) and 3-day Pi-deficient (–Pi) (**b**,**d**) conditions. Each dot represents an individual gene entity. Red and green dots represent, respectively, the PSi and PSr genes with differential regulation during Pi starvation in the WT (≥ 2-fold change in the shoot and ≥1.5-fold change in the root). Venn diagrams in each panel indicate the overlaps of the genes differentially expressed in the *PHT5;1-GFP*-overexpressing line compared with WT and PSR genes of WT. It is noteworthy that the WT PSi genes were upregulated in the *PHT5;1-GFP*-overexpressing line under +Pi conditions but downregulated under −Pi conditions. In a similar manner, the WT PSr genes were downregulated in *PHT5;1-GFP*-overexpressing line under +Pi conditions but upregulated under −Pi conditions. (**e**) Heat map analyses show the expression of a subset of Pi-starvation-upregulated genes with significant gene ontology (GO) enrichment in the category of root development, which were downregulated in the roots of the *PHT5;1-GFP*-overexpressing line. *P*-value was adjusted by Bonferroni correction.

We defined PSR genes as differentially expressed genes, either upregulated or downregulated when setting the *P*-value<0.05 based on the analysis of edgeR^37^ and the change of RPKM (reads per kilobase of exon model per million mapped reads) ≥2-fold in the shoot or ≥1.5-fold in the root of WT plants subjected to Pi starvation for 1 or 3 days. Under Pi-sufficient conditions, the gene expression patterns of two *PHT5;1-GFP-*overexpressing lines showed a positive correlation (Pearson's coefficient of correlation *r*=0.73) (Supplementary Fig. 6a). Compared with the WT, the differentially expressed genes in these two lines largely overlapped (Supplementary Fig. 6b). Taking the stronger *PHT5;1-GFP-*overexpressor 12 for further analyses, we found that 65.1 and 17% of the WT PSi genes (the PSR genes induced by Pi starvation in WT) were upregulated, respectively, in the shoot and root of the *PHT5;1-GFP* overexpressor under Pi-sufficient conditions (Venn diagrams in Fig. 6a,c). Up to 52.3 and 69.1% of the genes upregulated in the overexpresser relative to the WT, in the shoot and root respectively, were also WT PSi genes (Venn diagrams in Fig. 6a,c). Moreover, 50.4 and 34.0% of genes downregulated in the overexpresser in the shoot and root, respectively were WT PSr (Pi starvation repressed) genes.

To discern the overall changes of WT PSR genes in the *PHT5;1-*GFP overexpressor, we compared the gene expression between the overexpressor and WT plants by scatter plots (Fig. 6a–d). Notably, there is a tendency that the *PHT5;1-GFP* overexpressor had a higher level of expression of PSi genes but a lower level of expression of PSr genes under Pi-sufficient conditions (Fig. 6a,c). Although the magnitude of the altered gene expression was more evident in the shoot than in the root, they showed a similar trend. Collectively, these results suggest that the overexpression of PHT5;1 triggers Pi starvation-activated transcriptional reprogramming.

Interestingly, under Pi-deficient conditions, we found that the expression level of several of the PSi genes was suppressed in the *PHT5;1-GFP* overexpressor 12, whereas the expression of several of the PSr genes was upregulated (Fig. 6b,d). Similar results were also observed for the *PHT5;1-GFP* overexpressor 4 (Supplementary Fig. 6c,d). This may be due to an increased capacity of vac-Pi to buffer the low cyt-Pi in the overexpressors. In addition, a subset of the PSi genes that were downregulated in the root of the *PHT5;1-GFP* overexpressors under Pi-sufficient conditions were significantly enriched in the gene ontology (GO) categories of the root development (for example, root morphogenesis, root epidermal cell differentiation and trichoblast differentiation) (Fig. 6e). However, these genes were upregulated to an extent comparable to the WT after 3 days of Pi starvation, implying that the internal low cyt-Pi level of the *PHT5;1-GFP* overexpressors alone was not sufficient to activate these genes. In support of this, the changes of root morphology under low Pi are mainly associated with the external Pi concentrations^38^,^39^. Nonetheless, the primary cause for the downregulation of these genes in the *PHT5;1-GFP* overexpressors under Pi-sufficient conditions is currently not clear.

## Discussion

Similar to other nutrients, Pi has different fates after uptake into the root: (i) it can enter the cytoplasmic pool for the biosynthesis of P-containing compounds or the regulation of protein function and signalling transduction; (ii) it can also be transported radially, passing through several cell layers of the root, towards the vascular bundle for the long-distance translocation to the above-ground tissues; (iii) alternatively, it can enter the vacuole for storage^40^. For decades, ^31^P-NMR spectroscopic monitoring of the exchange of Pi between the cytoplasm and the vacuole suggested the existence of vacuolar transport systems^41^,^42^,^43^,^44^,^45^. However, the mechanism underlying the passage of Pi across the tonoplast remained obscure. Herein we identified the *Arabidopsis* SPX-MFS family as the long-sought-after vac-Pi importer and designated them as PHT5 Pi transporters, by phenotypic and functional characterization of the respective *PHT5* mutants and overexpressing lines.

Using *in vivo*^31^P-MRS analysis, we determined the subcellular compartmentation of Pi between the cytoplasm and the vacuole of the *Arabidopsis* seedlings. Under Pi-replete conditions, the total Pi levels of the single *pht5;1* and the triple knockouts were reduced by 40% relative to the WT plants (Fig. 2a,b); yet, only the triple mutant displayed an increased cyt-Pi level (Fig. 4). In line with the role of PHT5 proteins in facilitating Pi import across the tonoplast, the *PHT5*-overexpressing lines overaccumulated Pi in the vacuole, thus leading to a decreased cyt-Pi level (Fig. 4). Except one report^43^, distinguishing the cytosolic Pi from the organelle Pi by ^31^P-NMR analysis has been technically difficult because of the low amount of Pi and the similar pH values of these compartments. We thus speculated that the cytosolic Pi of the *PHT5;1*-overexpressing lines is even lower than the cyt-Pi level we measured.

Despite the partial redundancy of the three *Arabidopsis* PHT5 proteins, loss of PHT5;1 rather than PHT5;2 or PHT5;3 led to severe leaf necrosis on Pi replenishment following Pi starvation (Fig. 2c), suggesting that PHT5;1 plays a major role in mediating the Pi import to the vacuole, preventing Pi accumulation in the cytoplasm accompanied by massive Pi uptake into the cell. Such a protection mechanism of vac-Pi sequestration is critical for plant growth under varying environmental Pi status and can explain the tolerance of the *pho2* mutant or other Pi overaccumulators to high amounts of cellular Pi^33^,^34^,^46^,^47^. By contrast, the *PHT5*-overexpressing lines displayed a higher vac/cyt ratio of Pi compartmentation, a decreased cyt-Pi level and a disturbance of cytoplasmic homeostasis that impaired the plant growth (Figs 3 and 4). Furthermore, given that the triple mutants are viable, we cannot exclude the possibility that plants can exploit other strategies bypassing the PHT5-mediated passage of Pi for their survival.

Although we anticipated to find an altered expression of *PHT5* gene*s* in response to varying Pi supply, in fact the steady-state level of *PHT5;1* and *PHT5;3* transcripts in the shoot was independent of the Pi status (Fig. 1m,n). Regulation of *PHT5* genes at the translational or posttranslational level are likely to be involved. In the Pi-starved root, the reduced expression of *PHT5;3* (Fig. 1n) would prevent Pi import into the vacuoles; however, the upregulation of *PHT5;1* disagreed with this view (Fig. 1m). In rice, *OsSPX-MFS1* and *OsSPX-MFS2* (Os02g45520) also showed distinct responses to Pi starvation^23^,^26^. On transfer to low-Pi medium, the *Vtc* genes were upregulated and the protein complex was accumulated in yeast vacuolar membranes, to facilitate polyP synthesis and import to the vacuole^35^,^48^. This led to a lower cyt-Pi level whose feedback stabilized Pi starvation responses for the growth adaptation^48^. We thus speculate that certain Pi-starved plant cells might increase the vac-Pi transport activity to fine-tune the expression of PSR genes by keeping the cyt-Pi level low. Indeed, the vacuoles isolated from Pi-starved barley mesophyll cells showed higher activity of Pi transport than those from Pi-replete cells^17^.

In rice, *OsSPX-MFS1* and *OsSPX-MFS2* are the targets of a PSi microRNA, *Os*miR827 (refs 23, 26). *At*miR827 is also induced by Pi starvation in *Arabidopsis*^49^ and potentially able to cleave the transcript of *AtPHT5;1*, although it was not experimentally validated. Instead, *At*miR827 targets another SPX domain-containing protein, NITROGEN LIMITATION ADAPTATION, which regulates the degradation of PHT1 proteins by ubiquitination-mediated endocytosis^50^. Interestingly, *AtPHT5;1* and *OsSPX-MFS2* have several transcript variants with different 5′-untranslated region lengths, resulting in differential targeting of miR827.

Our initial attempt to express the *Arabidopsis* PHT5;1 in yeast cells was unsuccessful, owing to mistargeting or instability of the protein. Instead, we confirmed the capability of the rice OsSPX-MFS1 homologue to transport Pi into yeast vacuoles (Fig. 5). We observed that the Pi uptake activities of the OsSPX-MFS1-expressing yeast vacuoles was uncoupled from H^+^ and not affected by the presence of ATP (Fig. 5b,c), suggesting that the vac-Pi import is mediated by facilitated diffusion along the electrochemical gradient. The Pi transport into vacuoles isolated from Pi-replete barley leaves was reported to be independent of ATP^17^. However, ATP stimulated the Pi uptake into the vacuoles isolated from Pi-starved barley leaves and *Catharanthus* cell culture grown under Pi-sufficient medium, hinting at the requirement of an electrochemical gradient generated by the H^+^ pump^17^,^51^. It is worth mentioning that we failed to detect a difference in the Pi transport activity of the *Arabidopsis* intact vacuoles between the WT and the *pht5;1* knockout plants. We speculated that unknown cytosolic components required to activate the vac-Pi transport are missing in this experimental system, or that the Pi influx of the isolated *Arabidopsis* vacuoles is very low and thus limiting a detailed study^51^.

While this study was under revision, an independent study also reported the role of PHT5;1, which they named VPT1, in mediating vac-Pi storage^30^. Complementary to our observation, the electrophysiological analysis of *VPT1-GFP*-expressing vacuoles suggested that VPT1 mediates the cytosolic Pi concentration-dependent Pi inward current and possesses the single channel-like characteristics^30^. This channel-like property may explain the ATP and H^+^-independent Pi influx of yeast vacuoles we observed. Liu *et al.*^30^ also reported that the *vpt1* mutant (*pht5;1-3* in this study) is more sensitive to high Pi stress when grown in hydroponic medium containing 6.5 mM Pi. However, we did not observe this phenotype, possibly because the concentration is much higher than the Pi-replete growth conditions we used (250 μM Pi). Under our growth conditions, *pht5;1-3* mutants showed a reduction of fresh weight by ∼20% compared with WT plants; however, this phenotype was independent of Pi concentrations and was not observed in the other two allelic mutants, *pht5;1-1* and *pht5;1-2*, and even in the *pht5;1-2 pht5;2 pht5;3* triple mutant. Nevertheless, all the loss-of-function mutants of *PHT5;1*, *pht5;1-1*, *pht5;1-2* and *pht5;1-3* displayed consistent phenotypes on Pi replenishment.

As the expression of the rice *OsSPX-MFS1* complemented the phenotype of *Arabidopsis pht5;1* mutants (Supplementary Fig. 4), the *Arabidopsis* PHT5 proteins and the rice homologue SPX-MFS1 appear to play a similar role in transporting Pi across the tonoplast. However, OsSPX-MFS3 (Os06g03860) was concluded to be a vac-Pi efflux transporter in a recent report based on the influx of Pi into the *Xenopus* oocytes in which OsSPX-MFS3 was mislocalized at the plasma membrane^27^. The overexpression of *OsSPX-MFS3* led to a decreased Pi level in the rice vacuoles^27^. This seems contradictory to our current findings (and those of Liu *et al.*^30^) of the role of the *Arabidopsis* PHT5 proteins in vac-Pi storage and sequestration. We then analysed the phylogenetic relationship between 47 SPX-MFS homologues in 13 different species and found that the eudicot and monocot SPX-MFS homologues fall into two different clades (Supplementary Fig. 7). Although the *Arabidopsis* and rice SPX-MFS homologues share 68–72% amino acid identity, the result suggests that the monocot and eudicot *SPX-MFS* genes may diverge before the speciation of monocot and eudicot plants, and evolve independently. However, whether the divergence is associated with functional variation (Pi export versus Pi import) requires further validation. Intriguingly, a substantial activity of Pi efflux out of the *OsSPX-MFS3*-expressing X*enopus* oocytes was also detected^27^, hinting the potential capability of OsSPX-MFS3 to transport Pi bidirectionally. How the biophysical properties of PHT5 proteins in these different types of cells contribute to the underlying transport mechanism requires further investigation.

In budding yeast *S. cerevisiae*, the activation of the *Pho* pathway via intracellular Pi sensing is at the core of Pi starvation programme^52^. Despite the unique mechanism of polyP synthesis and storage in the yeast vacuole^35^,^36^, both the Pi fluxes at the plasma membrane and at the tonoplast control the amount of cyt-Pi^52^,^53^. Molecular mechanisms underlying Pi sensing, signalling and adaptation in multicellular organisms such as plants are even more complex^5^. Identification of the plant vac-Pi importers allowed us for the first time to address the effect of the internal Pi level on the global transcript expression. From the results of RNA-seq analyses, we propose that the low cyt-Pi level of the *PHT5;1*-overexpressing lines induces an internal signal of Pi starvation, triggering the genome-wide transcriptional reprogramming resembling the Pi starvation responses occurring in WT plants. These data suggest that the cyt-Pi level regulates the majority of PSR genes. Although most of the PSR genes were activated in the *PHT5;1*-overexpressing lines, the expression of a subset of PSR genes associated with trichoblast differentiation was not upregulated but even suppressed, raising the possibility that when the external Pi is replete, PHT5 proteins play a role in repressing the development of root-hair cells besides their involvement in the maintenance of cyt-Pi homeostasis.

Besides PHT5 proteins, the other eukaryotic SPX domain-containing Pi transporters such as the yeast Pho87, Pho90, Pho91 and the plant Pi exporter PHO1 are also key regulators of Pi homeostasis^19^,^21^,^22^. In yeast, the SPX domain of Pi transporters plays an inhibitory role in regulating the Pi transport activities through the interaction with the regulatory protein Spl2 (ref. 21). Although many SPX domain-containing proteins were shown to exhibit intrinsic properties required to interact with other proteins^21^,^50^,^54^,^55^,^56^,^57^, the elucidation of their structural–functional relationship and the identification of novel SPX domain-interacting proteins would be necessary to reveal how the additional cytoplasmic SPX domain modulates the transport activity of PHT5 proteins. Functional studies of different truncated and point mutation variants of PHT5 proteins may reveal more relevant information about their mode of regulation on a molecular basis.

In summary, identification of the *Arabidopsis* PHT5 family as the vac-Pi importers opens up a new area of investigation into how plants coordinate the vac-Pi transport activity with the cellular metabolism and environmental fluctuations. Our findings also invoke interesting questions regarding how Pi is translocated out of the vacuole for metabolic needs on Pi deficiency and during Pi remobilization. The rice OsSPX-MFS3 was recently shown to be responsible for the vac-Pi export^27^; however, our results here support that its paralogue (OsSPX-MFS1) and orthologues (*Arabidopsis* PHT5 proteins) contribute to the Pi import into vacuoles. In the future, identification of both Pi influx and efflux systems in the same plant species can offer clear and insightful information.

## Methods

### Growth conditions and plant material

*Arabidopsis thaliana* ecotype Columbia (Col-0) was used in this study. The *pht5;1-1* (SAIL_789_E03), *pht5;1-2* (SAIL_96_H01), *pht5;1-3* (SALK_006647), *pht5;2* (SALK_009309) and *pht5;3* (SAIL_422_D07) T-DNA insertion mutants were obtained from the Arabidopsis Stock Center. Seeds were surface sterilized and germinated on agar plates with one-half modified Hoagland nutrient solution containing 250 μM KH_2_PO_4_, 1% sucrose and 1% bactoagar. The Pi-sufficient (+Pi) and Pi-deficient (−Pi) media were supplemented with 250 and 10  μM KH_2_PO_4_, respectively, unless specified otherwise. For hydroponic growth, the medium was prepared without supplement of sucrose. For the plant samples used for ^31^P-MRS analysis, 20 mg of seeds were surface sterilized and evenly distributed on the surface of the 100-μm nylon mesh cell drainer (BD 352360) by mixing with sterilized 1% agarose. The seeds were germinated in the sterilized six-well culture plates, each containing 5 ml of one-half modified Hoagland nutrient solution with 1 mM KH_2_PO_4_ and 1% sucrose. Three days after germination, the growth medium was exchanged at 12–24 h intervals. For RNA-seq, sample preparations are described as follows: 10-day-old seedlings were germinated and grown under +Pi conditions on the agar plates and then transferred to the −Pi media for an additional 1–3 days.

### Gene constructions and plant transformation

All the insert fragments of interest were amplified and cloned into pCR8/GW/TOPO (Invitrogen) for sequencing and then recombined into the desired Gateway destination vectors via LR Clonase enzyme mix (Invitrogen). For *Arabidopsis* overexpression experiments, the open reading frame (ORF) of *PHT5;1*, *PHT5;3* and *OsSPX-MFS1* (Os04g48390) complementary DNA and *PHT5;2* from genomic DNA were cloned and recombined into the Gateway destination vector pK7FWG2.0 or pMDC32, designated as *p35S:PHT5;1-GFP*, *p35S:PHT5;3-GFP*, *p35S:GFP-OsSPX-MFS1* and *p35S:PHT5;2-HA*. For the promoter assay, *pPHT5:GUS* constructs were generated using pMDC162 as the Gateway destination vector. For the complementation of *pht5;1-3* mutants, the *PHT5;1* genomic sequence was cloned and recombined into the Gateway destination vector pGWB504. Transgenic plants were generated using an *Agrobacterium tumefaciens* dipping procedure^58^ via strain GV3101.

### Transient expression

Transformation of *Arabidopsis* mesophyll protoplasts for transient expression of fluorescence fusion proteins was performed with tape-*Arabidopsis* sandwich method^59^. The Agrobacterium-mediated transient expression in tobacco leaves was conducted^55^. *Agrobacterium* EHA105 strain harbouring the constructs of genes of interest was grown in Luria–Bertani medium, collected and resuspended in the infiltration medium (10 mM MgCl_2_, 10 mM MES and 100 μM acetosyringone) to an OD_600_ of 1.0. The second or third true leaves of 3-week-old tobacco plants was infiltrated with *Agrobacterium* and analysed at 3 days post infiltration. Confocal microscopy images were taken using a Zeiss LSM 510 META NLO DuoScan with objectives LCI Plan-Neofluar × 63/1.3 Imm and Plan-Apochromat × 100/1.4 oil. Excitation/emission wavelengths were 488 nm/495–510 nm for GFP.

### GUS staining

GUS activity was detected by a modified method^60^. Briefly, transgenic plants were vacuum infiltrated with solution containing 1.9 mM X-Gluc (5-bromo-4-chloro-3-indoyl-*β*-D-glucuronide) for 4 to 6 h and chlorophyll was then cleared from the sample by 75% ethanol after staining. At least ten independent transgenic lines for each construct were examined. Representative results were shown.

### Measurement of phosphate contents

Tissues were homogenized with 1% glacial acetic acid. After centrifugation, the supernatant was collected and mixed with assay solution containing 0.35% NH_4_MoO_4_, 0.86 N H_2_SO_4_ and 1.4% ascorbic acid. Pi contents were determined by colorimetric assay based on the formation of phosphomolybdate followed by its reduction with ascorbic acid^31^.

### RNA isolation and qRT–PCR

Total RNA was isolated using RNAzol (Molecular Research Center) followed by treatment with DNase I (Ambion), to eliminate genomic DNA contamination. cDNA was synthesized from 0.5 to 1 μg total RNA using Moloney murine leukemia virus reverse transcriptase (Invitrogen) with oligo(dT) primer. qRT–PCR was performed using the Power SYBR Green PCR Master Mix kit (Applied Biosystems) on a 7300 Real-Time PCR system (Applied Biosystems) according to the manufacturer's instructions. Relative expression levels were normalized to UBQ10 as an internal control. For RNA-seq, RNA quality was verified by Agilent Bioanalyzer 2100 and only samples with RNA integrity numbers above 9.0 were used. Oligonucleotide sequences of the primer pairs used for PCR and cloning are shown in Supplementary Table 1.

### High-throughput RNA-seq analysis

Two biological replicates per condition and about 20 seedlings per replicates were used for library synthesis. Twelve libraries were multiplexed and loaded on each lane of the Illumina Hiseq flow cell v3. Sequencing was then performed on a Hiseq 2500, as a 2 × 100 paired-end run, according to the manufacturer's protocol (Illumina). The trimmed paired reads from each sample were mapped against the *A. thaliana* reference (TAIR10) using CLC Genomics Workbench (CLC Bio, http://www.clcbio.com), in which the expression values were reported as RPKM. An average of 96% of trimmed reads equivalent to 17–33 million reads per library was successfully mapped to the genome. For statistical analysis, the software package edgeR (empirical analysis of DGE in R)^37^ implanted in CLC Genomics Workbench was employed. The exon mapped reads for individual gene were compared and false discovery rate-corrected *P*-values was calculated. Total count filter cutoff is set at 5 using the Estimate tag-wise dispersions. The entities with RPKM value <0.25 were assigned to 0.25 for calculation of fold changes. For differential expression comparisons, false discovery rate-corrected *P*-value <0.05 coupled with fold changes of RPKM was applied to filter out differentially expressed genes.

### ^31^P-MRS analysis

^31^P-MRS data were acquired using a 7T MRI system (ClinScan 70/30 USR, Bruker, Ettlingen, Germany) with a dual tuned 1H/31P Radiofrequency coil. Eight-day-old seedlings together with the cell drainer were rinsed with deionized distilled water. Next, the whole sample was immersed under 20 ml perfusion buffer (10 mM MOPS pH=7.5, 50 mM glucose and 0.1 mM CaSO_4_) in a glass beaker and then placed above the centre of the coil. One hundred microlitres of 25 mM methylene diphosphonate (MDP) was used as an internal reference of chemical shift (*δ*), the value corresponding 17.9 p.p.m. relative to 85% phosphate solution (0 p.p.m.). The positioning of plant samples, MDP reference and Radiofrequency coil were kept consistent among all the experiments.

Before the acquisition of phosphorus data, magnetic field homogeneity was shimmed over 25 × 25 × 20 mm^3^ volume based on the ^1^H signal from the sample. To ensure the homogeneity of the magnetic field, the shimming procedure was repeated until the linewidth of the water peak was <10 Hz. The shimming setting was then used for acquisition of phosphorus spectroscopic data. ^31^P data were collected using a single hard pulse sequence with free induction decay acquisition with the following parameters: bandwidth=6,002 Hz, repetition time (TR)=2,000 ms and number of averages=2,048. The scanning time on the ^31^P spectroscopy of each sample was 68.3 min. To examine the pH values of the observed vacuolar and cyt-Pi peaks, a pH calibration curve was obtained by using a series of 1 M phosphate solutions with various pH values from 4.8 to 7.4. All ^31^P data were quantified using the time-domain fitting software (jMRUI)^61^ with the AMARES algorithm. Spectral process included 35 Hz Gaussian apodization, automatic zero-order phase adjustment and the first-order phase adjustment with range fixed within −1 and 1. The resonances corresponding to the peak of vac-Pi (0.82–1.27 p.p.m.) and the cyt-Pi (cyt-Pi, 2.08–2.62 p.p.m.) were quantified. The ratios of vac-Pi to cyt-Pi were calculated based on jMRUI quantification results.

### *OsSPX-MFS1* expression in yeast and vacuole transport assay

The ORF of *OsSPX-MFS1* was cloned and recombined into the Gateway destination vector pAG415GPD-ccdB and transformed into *S. cerevisiae* strain *vtc4* mutant^36^. ^33^P transport assay was performed with isolated yeast vacuoles according to a modified protocol^62^. After incubation with ^33^Pi, vacuoles were floated and purified with silicon oil^63^ followed by radioactivity measurement. When applied, a final concentration of 4 mM ATP or 10 μM carbonyl cyanide m-chlorophenyl hydrazone (CCCP) was added in the uptake solution. The ^33^Pi uptake value at the zero-time point was used for the subtraction of background. To analyse the subcellular localization of OsSPX-MFS1, the ORF of *OsSPX-MFS1* without a stop codon was cloned into pAG415GPD-ccdB-eCFP, to fuse with enhanced cyan fluorescent protein (ECFP) at the C terminus. The fluorescent signal was observed by confocal microscopy.

## Additional information

**Accession codes:** The RNA-seq data have been deposited in the NCBI Gene Expression Omnibus under accession code GSE74856.

**How to cite this article:** Liu, T.-Y. *et al.* Identification of plant vacuolar transporters mediating phosphate storage. *Nat. Commun.* 7:11095 doi: 10.1038/ncomms11095 (2016).

## Supplementary Material

Supplementary Information Supplementary Figures 1-7 and Supplementary Table 1

Supplementary Data 1 RNA-seq analyses of WT and *p35S:PHT5;1-GFP*-overexpressing plants grown under Pi-sufficient and -deficient conditions.
