# Supplementary material for: Identification of plant vacuolar transporters mediating phosphate storage
Source: Nat Commun. 2016 Mar 31;7:11095. doi: 10.1038/ncomms11095 (PMC4821872; doi:10.1038/ncomms11095)
Supplement: Supplementary Information — Supplementary Figures 1-7 and Supplementary Table 1 [file ncomms11095-s1.pdf]

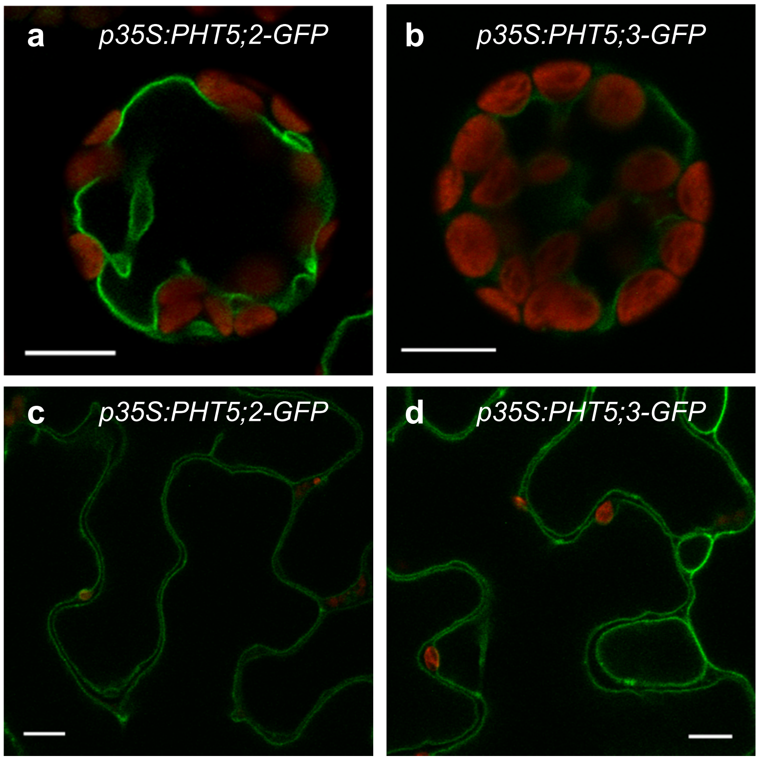

Supplementary Figure 1 | **Subcellular localization of *Arabidopsis* PHT5;2 and PHT5;3**
Expression of *p35S:AtPHT5;2-GFP* and *p35S:AtPHT5;3-GFP* in *Arabidopsis* mesophyll protoplasts (**a**,**b**) and *tobacco* (*Nicotiana benthamiana*) leaves (**c**,**d**). Scale bar, 10 µm.

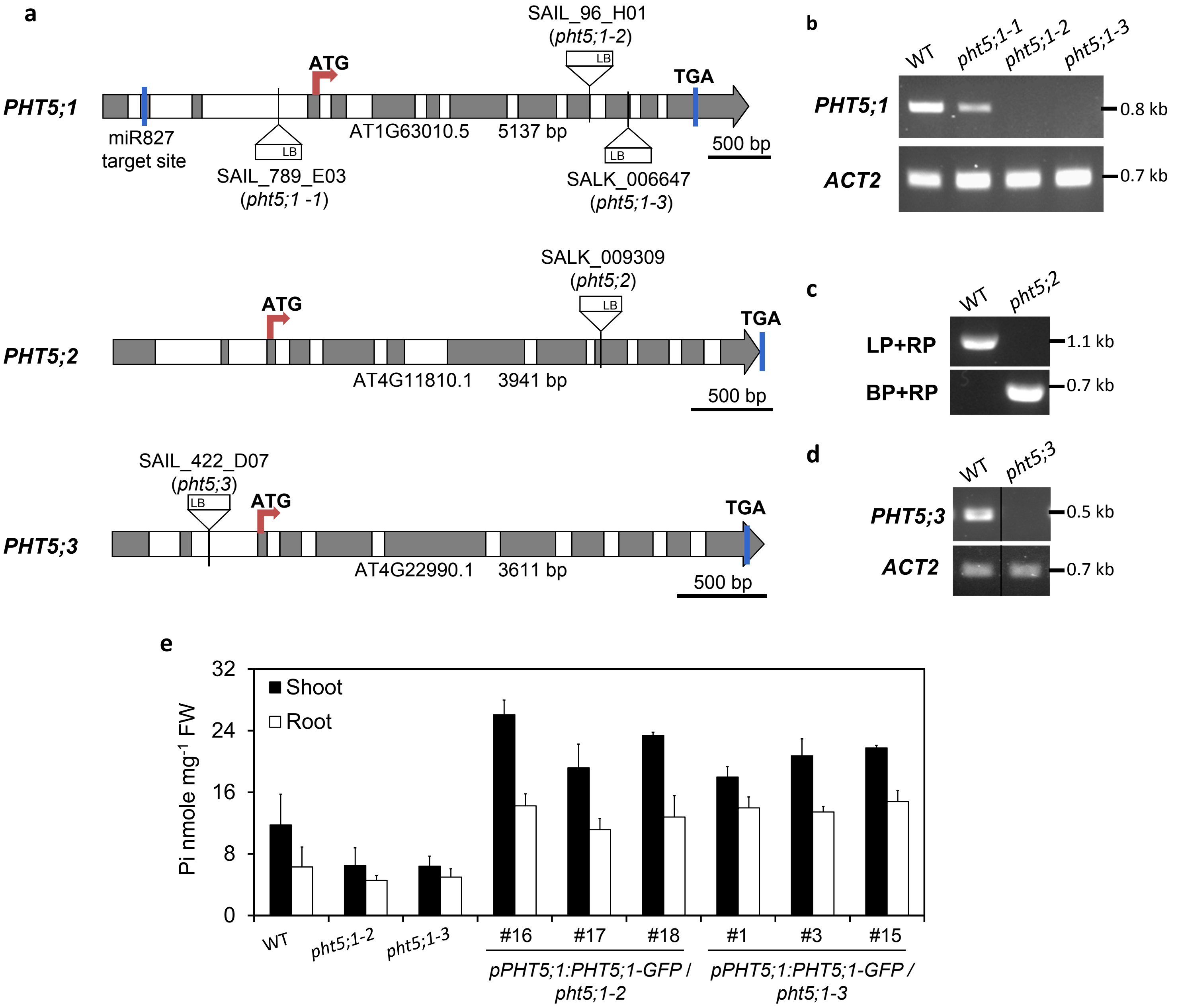

Supplementary Figure 2 | Characterization and complementation of the mutants of the *PHT5*s

(**a**) Schematic representation of T-DNA insertion in the mutants of PHT5s.

(**b,d**) RT-PCR analyses of *PHT5;1 and PHT5;3* transcripts in the respective mutants of *PHT5s*. (**c**) Because of extremely low expression of *PHT5;2*, PCR analysis of genomic DNA was employed to confirm homozygous T-DNA inserted mutation at *PHT5;2* locus (**e**) Complementation of the *pht5;1-2* or *pht5;1-3* mutant by the expression of *PHT5;1-GFP* driven by its native promoter. Three independent transgenic lines in each mutant background are shown. Error bar, s.d. (*n*=3).

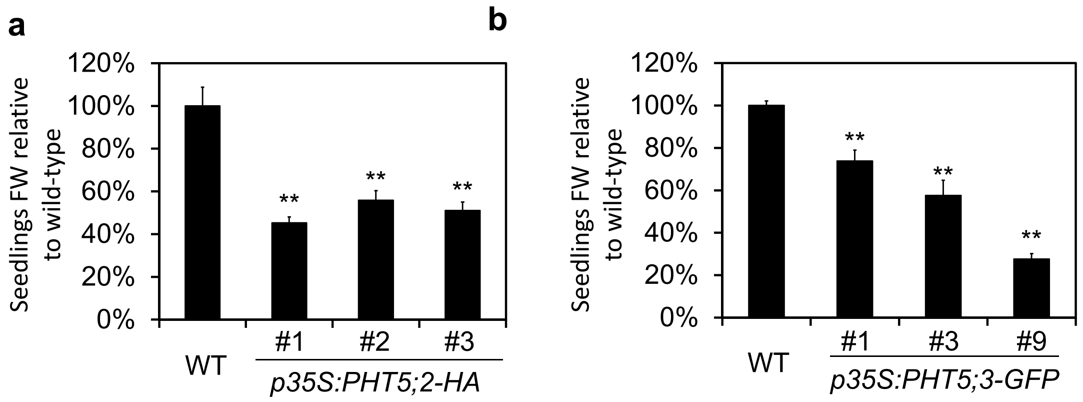

Supplementary Figure 3 | Overexpression of *Arabidopsis PHT5;2* and *PHT5;3* leads to retarded growth
The relative fresh weight of seedlings of *PHT5;2-HA*-overexpressing lines (11-day-old, **a**) and *PHT5;3-GFP*-overexpressing lines (12-day-old, **b**) to the corresponding wild-type plants (WT). Three independent transgenic lines are shown for each construct. Error bar, s.d. (n=3), *P<0.05, **P<0.01, Student's t-test. Results were reproducible in at least three independent experiments.

a

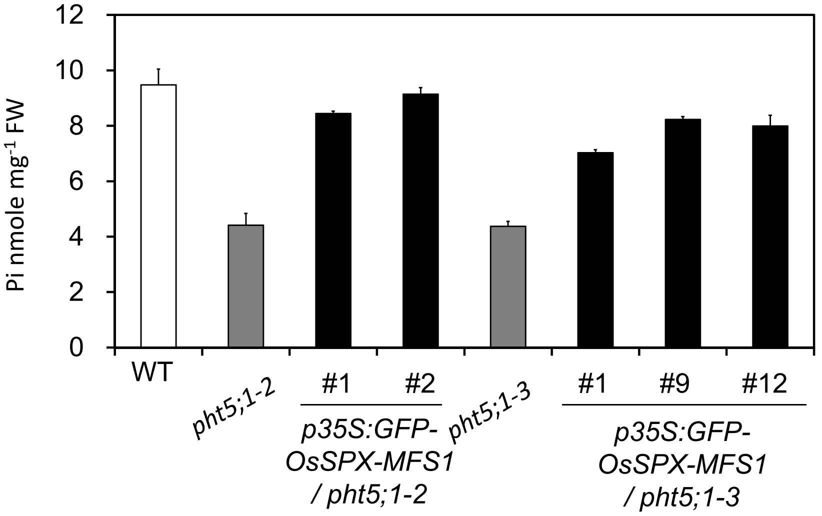

b

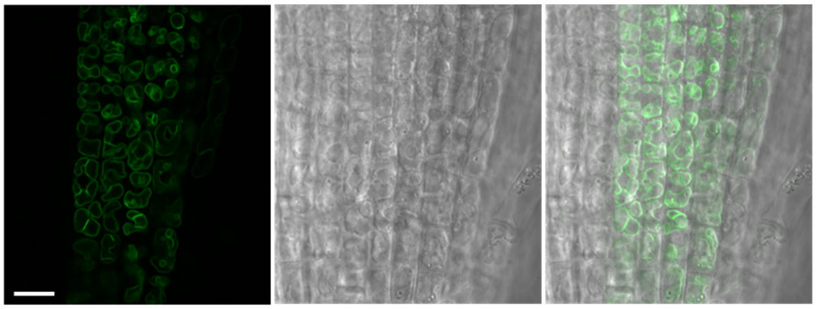

Supplementary Figure 4 | **Characterization of function and sub-cellular localization of OsSPX-MFS1 in *Arabidopsis***
(**a**) Pi concentrations of the first leaf of 14-day-old *Arabidopsis* wild type (WT), *pht5;1-2* and *pht5;1-3* plants and *p35S:GFP-OsSPX-MFS1*-overexpressing lines grown under +Pi conditions. Two-three independent transgenic lines in each mutant background are shown. Error bar, s.d., technique replicates *n*=2. (**b**) Fluorescence signal (left), bright field (middle) and merged image (right) of *pht5;1-3* mutant transformed with a *p35S:GFP-OsSPX-MFS1* construct. Scale bar, 10 µm

Log_2(FC): -2 to 2

	PAP17	At4	IPS1	SPX1	SPX3	miR399b	miR399d	PHF1	PHT1;2	PHT1;4	PHT1;9	PHT3;2	PHO1;H1	G3PP1	SQD1	MGD3	ATGOLS2	**Average**
pht5;1-2	-0.6	0.6	-0.2	0.0	-0.8	-0.4	0.8	-0.4	0.3	-0.1	1.2	0.8	0.0	0.1	-0.3	-0.1	0.2	0.1
pht5;1 pht5;2 pht5;3	-0.3	-0.1	-0.6	-0.1	-0.4	-0.8	0.9	-0.8	-0.8	-0.4	0.4	0.7	-0.9	0.2	-0.6	-0.7	-1.8	-0.4
p35S:PHT5;1-GFP #04	1.8	0.7	-0.2	0.8	0.8	-0.4	0.3	1.8	0.7	0.8	0.4	2.6	1.0	1.7	0.2	0.2	1.7	0.9
p35S:PHT5;1-GFP#12	2.8	-0.1	-0.8	1.2	3.0	-0.4	-0.2	1.5	0.5	1.6	0.9	4.8	1.2	3.2	0.0	-0.1	2.6	1.3
WT_Pi deficient	6.5	5.9	2.5	4.2	6.8	2.1	6.3	1.7	1.0	2.4	3.0	1.9	5.7	5.4	2.3	4.0	5.9	4.0

Supplementary Figure 5 | Overexpression of *PHT5;1* leads to the upregulation of Pi starvation-induced genes under Pi-sufficient conditions.

Heat map of qRT-PCR results reveals the upregulation of many Pi starvation-induced (PSi) genes in the shoot of 10-day-old *PHT5;1*-overexpressing lines, implying that a lower cyt-Pi level of the *PHT5;1*-overexpressing lines likely induced Pi-starvation responses. In contrast, these PSi genes were downregulated in the *pht5* triple mutants. The numbers indicate the log_2 values of fold changes of gene expression relative to WT plants grown under Pi sufficiency. Results were reproducible in three independent experiments.

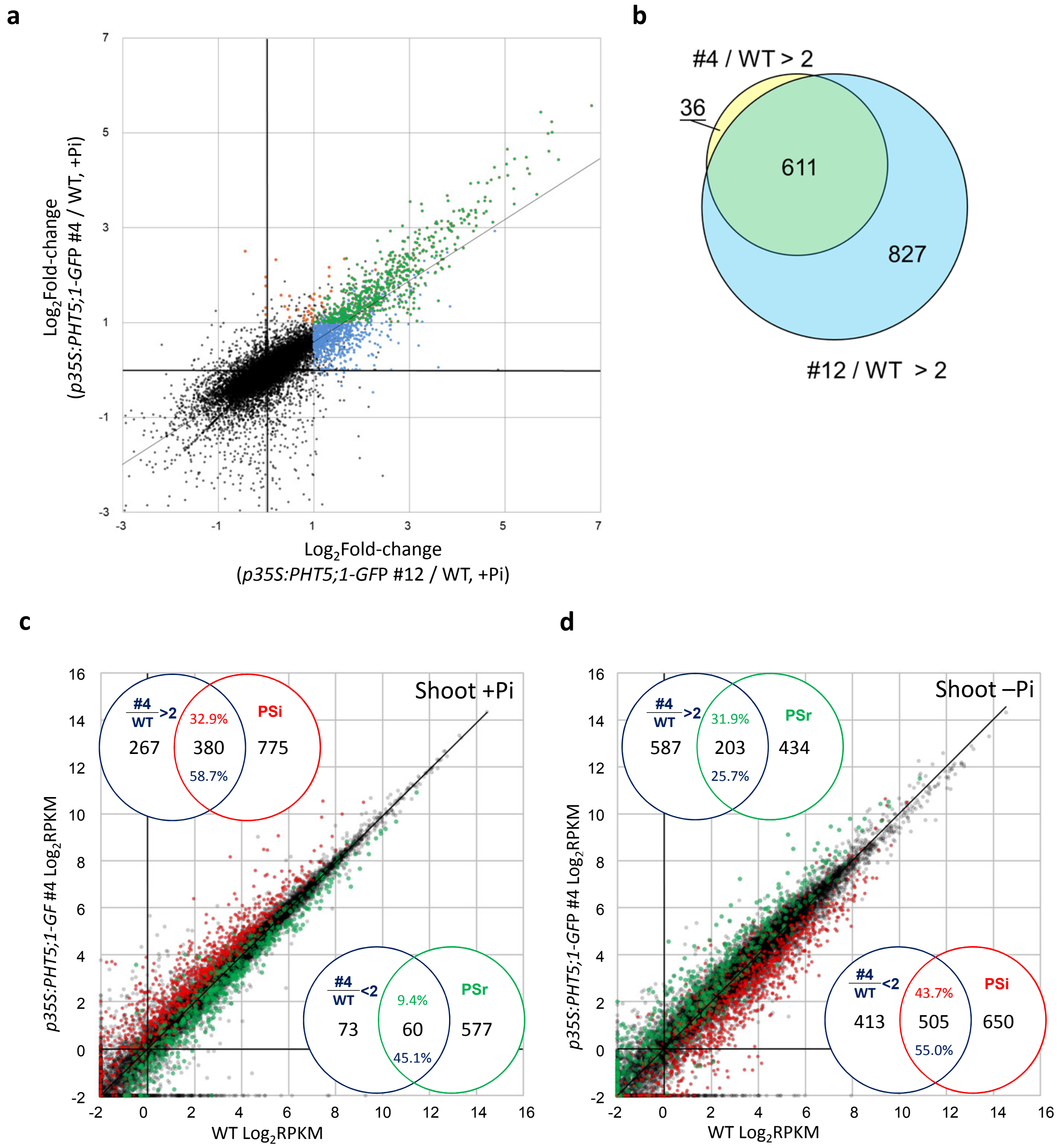

Supplementary Figure 6 | Overexpression of *PHT5;1* leads to mis-regulation of PSR genes.
(**a**) The positive correlation of the gene expression pattern between two *PHT5;1*-overexpression lines, #4 and #12, grown under Pi-sufficient conditions (Pearson coefficient of correlation r = 0.73). (**b**) Venn diagram showing the overlap of differentially expressed genes in the *PHT5;1*-overexpressing lines #4 and #12.
Scatter plots showing the relationship of the expression of an individual gene (a single dot) in the shoots between WT and *PHT5;1* overexpressing plants (line #4) grown under Pi sufficient medium (**c**) or starvation for 3 days (**d**). The Pi-starvation-upregulated and -downregulated genes are marked in red and green, respectively. The Venn diagrams in each patterns indicate the proportion of upregulated or downregulated genes in *PHT5;1* overexpressing lines belonging to PSR genes of WT.

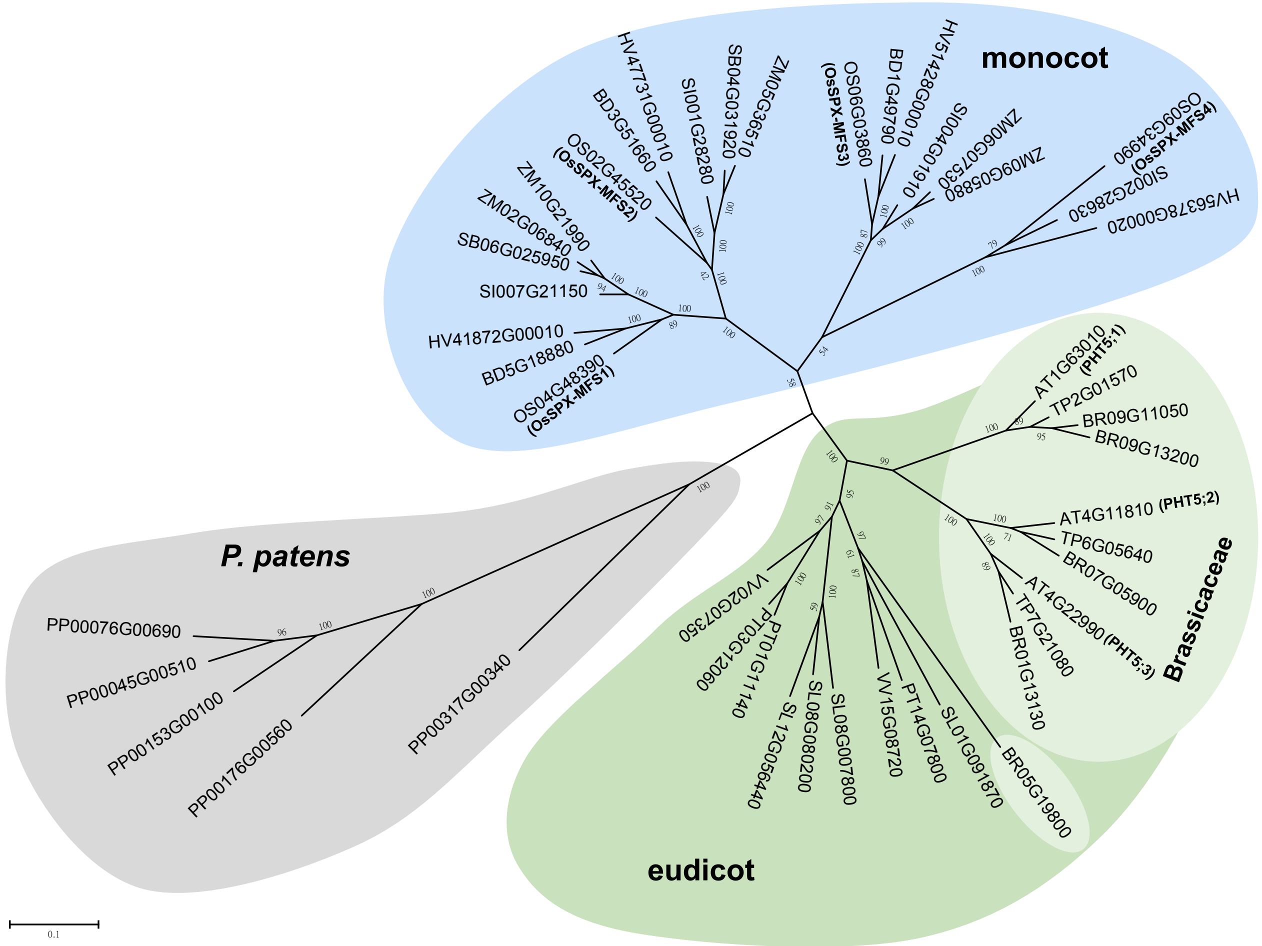

Supplementary Figure 7 | **Phylogenetic analysis of *SPX-MFS* homologs.**
The coding DNA sequences of 47 *SPX-MFS* homologs in 13 different species obtained from PLAZA 3.0 (http://bioinformatics.psb.ugent.be/plaza/) were aligned using ClustalW. The phylogenetic tree was built based on Maximum Likelihood with the Tamura-Nei model. The scale bar indicates genetic distance of branch length measured in the number of nucleotide substitutions per site. *Oryza sativa* japonica (OS), *Sorghum bicolor* (SB), *Zea mays* (ZM), *Setaria italic* (SI), *Brachypodium distachyon* (BD), *Hordeum vulgare* (HV), *Arabidopsis thaliana* (AT), *Brassica rapa* (BR), *Thellungiella parvula* (TP), *Populus trichocarpa* (PT), *Vitis vinifera* (VV), *Solanum lycopersicum* (SL) and *Physcomitrella patens* (PP).

Supplementary Table 1 | List of primers

Gene		Sequences (5'→3')
**Q-RT-PCR**		
AT1G63010	Forward	TAACCAATCTACTTTGCCGGGAT
PHT5;1	Reverse	CTCTGAATGAAATGCATAGCCATAC
AT4G22990	Forward	GGATCGGCGAGAGCAGTGAA
PHT5;3	Reverse	CTTTAACGGTACACAGTCGCTTATAT
AT4G05320	Forward	GGCCTTGTATAATCCCTGATGAATAAG
UBQ10	Reverse	AAAGAGATAACAGGAACGGAAACATAGT
AT3G17790	Forward	TTGTTGACACGACTCCTTTCGT
PAP17	Reverse	ATCACGGAGAAGAGCTTTGACAT
AT5G03545	Forward	AACCCTAACAACATCCATTGTTGAA
AT4	Reverse	GAAACTTGAACCTCTCAAAACCCT
AT3G09922	Forward	TTTGGAGAATAGTCAGACCAGTGC
IPS1	Reverse	TCACTATAAAGAGAATCGGAAGCA
AT5G20150	Forward	GATTCCATTGTTGGAGCAAGA
SPX1	Reverse	AATCTGTTAGCTTCTTCTATTGTA
AT2G45130	Forward	CCGATCTCTTCATCTATCTT
SPX3	Reverse	TTACGATAATGTCATATTGCGT
AT1G63005	Forward	AGGTCCTTTACTTCCAAATATACACATACA
MIR399B	Reverse	TCGATCATCGGAAATTTTCGA
AT2G34202	Forward	TTACTGGGCGAATACTCCTATGG
MIR399D	Reverse	ATTTTACTTGCATATCTAGCCAATGC
AT3G52190	Forward	GCCTTCTGAGGATCATAGTAG
PHF1	Reverse	CGCTGCAACAAGATAAGGAAG
AT5G43370	Forward	AGCCATCATTGGAGCCTTC
PHT1;2	Reverse	ACCTTAGCCTTGTCTTGATT
AT2G38940	Forward	GGTCCCAATAGTTTAGGTGAT
PHT1;4	Reverse	AGTTGCTAGAGACAAGGAGAA
AT1G76430	Forward	GAAGATCGTTAGAAGAGAACGAA
PHT1;9	Reverse	GTATTGTCTCCGAAGTAACTCA
AT3G48850	Forward	GCTCCATCTGTAAGTGCATA
PHT3;2	Reverse	CTGGTAGTTCTGTTTCCCTT
AT1G68740	Forward	GATGAAGAAGACTAATCTGATC
PHO1;H1	Reverse	GTTCTTTCAAATCTGACGAAGC
AT3G47420	Forward	AGGCGAATATTTATCGGACGAA
G3PP1	Reverse	ACACCTCCTACATCGAACATTGTC

Gene		Sequences (5'→3')
AT4G33030	Forward	CATTGACTCCTATTGCCTCCATT
SQD1	Reverse	TCCCTGTCAAAGCCTTCCAT
AT2G11810	Forward	TGCGGCCGGAACAAAG
MGD3	Reverse	CCTTGACCGGAATCTTCCATT
AT1G56600	Forward	AATATAATCATCGAGCTCGT
GOLS2	Reverse	TTATAGTCATGAAGAGGCG
**RT-PCR**		
AT3G18780	Forward	GTAGTCAACAGCAACAAAGGAGAGC
ACTIN2	Reverse	ATGGCTGAGGCTGATGATATTCA
AT1G63010	Forward	TTGTGAGTGCAAGTGCTCTTG
PHT5;1	Reverse	TACCCGCGATAACAACGATAG
AT4G22990	Forward	TCGTGTGATCCCCTTAGTGTC
PHT5;3	Reverse	TTTCCTTTCTCACCGTATCCC
**CONFIRM T-DNA**		
SAIL_BP	BP	TAGCATCTGAATTTCATAACCAATCTCGATACAC
SALK_BP	BP	TGGTTCACGTAGTGGGCCATCG
pht5;1-1	LP	TTTTCACTTGAAATGCACGAG
	RP	GAAATCTTTGAGAACATGGCG
pht5;1-2	LP	TTGTGAGTGCAAGTGCTCTTG
	RP	TACCCGCGATAACAACGATAG
pht5;1-3	LP	TACCCGCGATAACAACGATAG
	RP	TTGTGAGTGCAAGTGCTCTTG
pht5;2	LP	GGTTTGTGGTGTTGTTATCGG
	RP	GACGCTAACAGAATTTGCCTG
pht5;3	LP	TCGTGTGATCCCCTTAGTGTC
	RP	TTTCCTTTCTCACCGTATCCC
**CONSTRUCTS**		
pPHT5;1:GUS	Forward	CTTTTAATCGCAGAAAGCAGAGAGC
	Reverse	CTGTTCTTTACCTTGTTTATATCCGTCT
pPHT5;2:GUS	Forward	CTTCTACACTGAAAGAAAGTCACAGAG
	Reverse	GGAGATGGATATGAGCAGATAGCTAATC
pPHT5;3:GUS	Forward	AGAAACGAATCACGTGGCTTTGTTTTC
	Reverse	TTTCTACAACAAAACAAGAATCAGAGAC
p35S:PHT5;1-GFP	Forward	ATGGTGGCTTTTGGGAAATACTTGCAGC
	Reverse	ATAGAGTGAGTTATAAGTACAACAAGTAGC
p35S:PHT5;2-GFP	Forward	ATGGTCGCCTTTGGAAAAAAGCTCAAGG
	Reverse	ATACAAGGAGTTATAAGTATAACAAGTAGC

Gene		Sequences (5'→3')
p35S:PHT5;2-HA	Forward	ATGGTCGCCTTTGGAAAAAAGCTCAAGG
	Reverse	TTAAGCGTAATCTGGAACATCGTATGGGTAATACAAGGAGTTATAAG
p35S:PHT5;3-GFP	Forward	ATGGTAGCCTTCGGGAAAAAACTCAAGG
	Reverse	ATACAAGGAGTTATAAGTAAAACAAG
pPHT5;1:PHT5;1-GFP	Forward	CTTTTAATCGCAGAAAGCAGAGAGC
	Reverse	TCAATAGAGTGAGTTATAAGTACAAC
p35S:GFP-OsSPX-MFS1	Forward	ATGGTTAATTTTGGAAAGAAGTTG
	Reverse	TTAGTACAGGGTGTTGTATGTG
OsSPX-MFS1-ECFP	Forward	ATGGTTAATTTTGGAAAGAAGTTG
	Reverse	GTACAGGGTGTTGTATGTGCAG
